# Hyperglycemia Increases Susceptibility to Ischemic Necrosis

**DOI:** 10.1155/2013/490964

**Published:** 2012-12-23

**Authors:** D. Lévigne, M. Tobalem, A. Modarressi, B. Pittet-Cuénod

**Affiliations:** Division of Plastic, Reconstructive and Aesthetic Surgery, University Hospitals of Geneva and Faculty of Medicine, University of Geneva, Rue Gabrielle-Perret-Gentil 4, 1211 Geneva 14, Switzerland

## Abstract

Diabetic patients are at risk for spontaneous foot ulcers, chronic wounds, infections, and tissue necrosis. Current theories suggest that the development and progression of diabetic foot ulcers are mainly caused by arteriosclerosis and peripheral neuropathy. Tissue necrosis plays a primordial role in the progression of diabetic foot ulcers but the underlying mechanisms are poorly understood. The aim of the present study was to investigate the effects of hyperglycemia *per se* on the susceptibility of ischemic tissue to necrosis, using a critical ischemic hind limb animal model. We inflicted the same degree of ischemia in both euglycemic and streptozotocin-induced hyperglycemic rats by resecting the external iliac, the femoral, and the saphenous arteries. Postoperative laser Doppler flowmetry of the ischemic feet showed the same degree of reduction in skin perfusion in both hyperglycemic and euglycemic animals. Nevertheless, we found a significantly higher rate of limb necrosis in hyperglycemic rats compared to euglycemic rats (71% versus 29%, resp.). In this study, we revealed that hyperglycemia *per se* increases the susceptibility to limb necrosis in ischemic conditions. Our results may help to better understand the physiopathology of progressive diabetic wounds and underline the importance of strict glycemic control in patients with critical limb ischemia.

## 1. Introduction

Diabetes mellitus (DM) favors the development of spontaneous foot and leg ulcers, chronic wounds, infections, and gangrene [1, 2]. Approximately 85% of amputations in diabetic patients are preceded by a foot ulcer, which subsequently deteriorates into a severe infection or gangrene [3]. These amputations significantly contribute to the high morbidity and mortality rates as well as the high health care costs in patients with diabetic foot ulcers [4, 5]. The most important factors related to the development of diabetic foot ulcers are peripheral neuropathy and arteriosclerosis. Furthermore, diabetic peripheral neuropathy not only leads to the loss of protective sensation, increasing the risk of unnoticed minor traumas, but also results in the degeneration of the sympathetic nerves innervating thermoregulatory arteriovenous shunt vessels of the skin [6]. These denervated shunt vessels lose their normal capacity of contraction and the nutritional skin capillaries are bypassed leading to the development of chronic capillary ischemia [7]. At the same time, DM is associated with an acceleration of arteriosclerotic changes of the greater arteries resulting in decreased blood flow, further contributing to a limb threatening ischemic condition [8].

However, it remains elusive whether hyperglycemia itself has a direct influence on the development of foot ulcers and its complications, especially in ischemic conditions. The aim of the present study was to evaluate the role of hyperglycemia *per se* in the physiopathology of spontaneous ischemic ulcers and on the susceptibility for ischemic necrosis, using a critical ischemic hind limb animal model with streptozotocin-induced hyperglycemic rats.

## 2. Methods

### 2.1. Animals

Male Wistar rats (*n* = 14) aged 90 days and weighing 300–350 g (Charles River Laboratories, L'Arbresle Cedex, France) were fed a standard diet and given water ad libitum. The local ethics committee and veterinary authority approved all procedures according to Swiss guidelines.

Rats were randomly assigned to two groups of equal size (*n* = 7): control group and hyperglycemic group. Hyperglycemia was induced by single i.p. injection of streptozotocin (STZ) (Sigma-Aldrich, St.Louis, MO, USA) three weeks before surgery. STZ was dissolved in sterile 0.1 M citrate buffer (pH 4.5) and injected intraperitoneally (50 mg/kg body weight) within 10 minutes of the preparation. Blood glucose levels were measured from tail venous blood by blood glucose test strips and just before sacrificing from carotid blood using glucose oxidase method (Glu; Roche Diagnostics, Rotkreuz, Switzerland). Rats with glucose levels >11.1 mM were included in the hyperglycemic group.

### 2.2. Ischemic Foot Model

**Figure 1:**
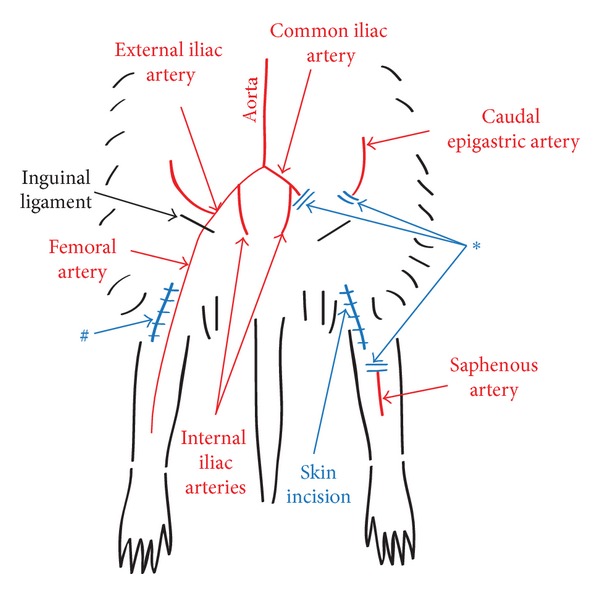
Ischemic foot model. *Ischemic limb: Resection of the external iliac, the femoral, and the saphenous arteries to the level of the knee. ^#^Nonischemic limb: dissection without resection of the above cited arteries.

As previously described [9], through a longitudinal incision in the inguinal region that was shaved, the external iliac and femoral arteries were dissected from the common iliac to the saphenous arteries. To provoke an ischemic condition, the dissected arteries were resected from the common iliac in the left limb (ischemic limb, Figure 1) while in the right limb arteries were conserved and limbs considered being nonischemic (sham surgery). All surgical procedures were performed under an operating microscope (Carl Zeiss, Jena, Germany), and animals were anesthetized by inhalation of isoflurane 5% for induction and 3% for maintenance of anesthesia.

### 2.3. Experimental Groups

We compared streptozotocin-induced hyperglycemic rats with euglycemic rats (*n* = 7 per group). By infliction of unilateral ischemia in both animal groups, we created four conditions: (1) hyperglycemic rat with nonischemic limb, (2) hyperglycemic rat with ischemic limb, (3) euglycemic rat with nonischemic limb, and (4) euglycemic rat with ischemic limb.

### 2.4. Macroscopic Limb Evaluation

Limbs were assessed macroscopically every two days during the first week and thereafter twice a week to evaluate necrosis status. For ethical reasons the animals were sacrificed as soon as limb necrosis was observed.

### 2.5. Percutaneous Laser Doppler Measurements

Skin blood flow was measured using a percutaneous laser Doppler perfusion monitor (PIM II Laser Doppler Perfusion Imager, LDPIwin 2.0.6 software, Lisca AB Berzelius Science Park, Linköping, Sweden). Measurements were carried out on the dorsal skin of the hind feet, immediately before and after surgery and during the observation period until sacrifice.

### 2.6. Statistical Methods

Data was analyzed with the use of Stata software, version 11.0. Statistical analysis consisted in a comparison of data from hyperglycemic versus euglycemic animals, in both ischemic and nonischemic conditions. Comparison of values of perfusion was performed using the nonparametric Kruskall-Wallis test followed by the measures to correct the *α*-error according to Bonferroni probabilities. Comparison of the proportions of limb necrosis was performed using Fisher's test. Differences were considered significant at *P* < 0.05.

## 3. Results

### 3.1. Blood Flow Measurement

Blood perfusion levels, measured by laser Doppler flowmetry, were similar in euglycemic and hyperglycemic animals in both ischemic and nonischemic conditions. In ischemic limbs the blood flow significantly decreased immediately after resection of the arteries, and in animals which did not present necrosis the blood flow remained low during the first week (Table 1).

### 3.2. Limb Necrosis

In nonischemic conditions, no foot necrosis was observed neither in euglycemic nor in hyperglycemic animals. In ischemic conditions, a significantly higher occurrence of limb necrosis was observed in hyperglycemic animals compared to euglycemic animals.

In hyperglycemic animals, five out of seven ischemic limbs showed total necrosis (71%), affecting the feet and lower limbs up to the level of the knee. This rate of limb necrosis was significantly higher than in ischemic limbs of euglycemic rats (2 out of 7, 29%, *P* < 0.025, Table 2).

In limbs that would eventually become necrotic, we observed signs of critical ischemia starting from day three (cyanotic aspect, stiffness, and coldness) that rapidly progressed in necrosis from proximal to distal, motivating the immediate sacrifice of the animal.

## 4. Discussion

Every 30 seconds, somewhere in the world, a lower limb is amputated due to DM [10]. The progression of critical ischemic limbs to necrosis and gangrene is reportedly 40% in diabetic patients compared to 9% in nondiabetic patients [11]. Since current therapeutic approaches of diabetic wounds often are unsatisfactory, it is of crucial importance to better understand the pathophysiology of the progressive necrosis at the wound margins that leads to the characteristic extension of diabetic wounds.

In one of our precedent studies, we developed a new model of ischemic foot ulcers. We aimed at creating a severe ischemic condition without provoking limb necrosis to study wound healing in ischemic conditions [9]. We assessed different degrees of ischemic conditions by resecting arteries on different levels. We concluded that in euglycemic rats, resection of the external iliac artery, the femoral artery, and the saphenous artery down to the level of the knee resulted in a severe ischemic condition with a delayed wound healing process, but with low occurrence of limb necrosis. 

In the present study, we aimed at assessing the effects of hyperglycemia on tissue susceptibility for ischemic necrosis. We used the same model as described above in euglycemic and streptozotocin-induced hyperglycemic rats. The same degree of ischemia was inflicted to euglycemic and hyperglycemic rats by resecting the external iliac, the femoral and the saphenous artery. To avoid the effects of long-term complications of DM, in particular of vasculopathy and neuropathy, we induced ischemia only three weeks after injection of streptozotocin. By laser Doppler flowmetry we confirmed that the skin perfusion at the dorsal surface of the feet was indeed comparable in hyperglycemic and euglycemic animals. Nevertheless, we found a significantly higher rate of limb necrosis in hyperglycemic rats compared to euglycemic rats (71% versus 29%, resp.).

Our results demonstrate a direct relation between hyperglycemia and acute ischemic necrosis. We hypothesize that the decrease of tissue tolerance to ischemia induced by hyperglycemia could be responsible for the initial skin lesions, which would lead to the development of diabetic ulcers. This could explain the specific evolution of diabetic wounds toward peripheral progressive necrosis. 

The phenomenon of decreased tolerance to ischemia caused by hyperglycemia has already been investigated in other ischemic tissues, notably in heart and brain [12–15]. Hyperglycemia has been associated with impaired outcome and expanded infarct volume in patients with ischemic strokes and myocardial infarctions [15, 16]. A recent meta-analysis of the association between hyperglycemia and stroke size in animal models revealed that hyperglycemic animals presented 94% larger infarct areas (140% in streptozotocin induced hyperglycemia and 48% after dextrose infusion) compared to euglycemic animals [17]. Several mechanisms have been identified through which hyperglycemia could aggravate cerebral and cardiac injury after ischemia, whereas a mechanistic link between hyperglycemia and susceptibility to necrosis is yet to be established. We propose the following hypotheses to explain how hyperglycemia *per se* could sensitize to ischemic tissue necrosis: (1) increased blood viscosity and hypercoagulability worsening progressively the level of ischemia, and (2) direct cellular toxicity due to the development of increased oxidative stress and lactic acidosis.

Hyperglycemia increases plasma fibrinogen levels and thereby blood viscosity and coagulability [18–20]. In nondiabetic patients with acute ischemic stroke, high glucose levels are related to a hypercoagulant state and a worsened outcome [21]. Furthermore, it has been shown that increased blood viscosity is associated with decreased tcPO2 levels in diabetic feet [22]. The increased blood viscosity in hyperglycemic conditions could therefore further aggravate the oxygen deprivation caused by the initial ischemia. In fact, it has been shown that dalteparin, a low molecule weight heparin, improves local tissue oxygenation in patients with diabetes, severe vascular disease, and foot ulcers [22].

Oxidative stress induced by hyperglycemia has been suggested as another possible mechanism of tissue injury exacerbation after strokes and myocardial infarctions [23, 24]. Indeed, both ischemia and hyperglycemia leads to excessive production of reactive oxygen species (ROS), and it is possible that the two factors accelerate each other. On the one hand, ischemia leads to oxidative stress through the generation of ROS through NADPH oxidase and other oxidases [25]. On the other hand, glucose has been shown to be the requisite electron donor for superoxide production by NADPH oxidase [23]. Additionally, hyperglycemia leads to the generation of free oxygen radicals also through the accumulation of advanced glycosylation end products (AGEs) [26, 27]. Further research is needed in order to evaluate whether the oxidative stress provoked by ischemia and exacerbated by hyperglycemia is a major player in the pathophysiology of ischemic cell necrosis.

Lactic acidosis could be another important contributor to ischemic cell death in hyperglycemic conditions [28]. When blood flow and oxygen supply decrease to critical levels, cells generate energy predominantly through anaerobic glycolysis. As a result, blood concentration of lactic acid rises and the pH level drops [29, 30]. Under hyperglycemic conditions, there is more substrate available for anaerobic glycolysis and consequently lactic acid production rises [31]. In fact, in an ischemic stroke animal model, it was shown that bolus infusion of glucose resulted in a severe intracellular acidosis and increased tissue death [32]. We suggest that when combined with elevated glucose levels, the ischemia-induced shift to anaerobic glycolysis leads to elevated tissue acidosis, which may accentuate cellular death related to ischemia.

In hyperglycemic conditions, the combination of oxidative stress, acidosis, and hypercoagulability creates a nocuous cellular environment that favors progressive ischemic necrosis. As the mechanisms through which both ischemia and hyperglycemia induce cellular toxicity are similar, it is possible that they amplify each other.

## 5. Conclusions

The unfavorable evolution of many diabetic feet is not only a result of neurogenic and arteriosclerotic long-term effects of diabetes, but could also be, as we observed, a direct effect of hyperglycemia itself. In this study, we demonstrated for the first time that at similar levels of ischemia, limb necrosis appears significantly more often in hyperglycemic conditions compared to euglycemic conditions. This finding suggests that hyperglycemia sensitizes to the effects of ischemia, revealing a direct involvement of hyperglycemia in the events leading to ischemic necrosis. Our results may help to better understand the physiopathology of progressive diabetic wounds and underline the importance of strict glycemic control in patients with critical limb ischemia.

## Figures and Tables

**Table 1:** Skin blood perfusion (PU) before (baseline) and after resection of the arteries or sham surgery (days 1 and 7), in euglycemic and hyperglycemic animals.

	Euglycemic animals		Hyperglycemic animals	
	Sham surgery	Resection	Sham surgery	Resection
Baseline	6.1 ± 0.4	5.8 ± 0.4	5.8 ± 0.3	6.5 ± 0.6
Day 1	6.0 ± 0.8	3.3 ± 0.1*	5.0 ± 0.5	3.4 ± 0.2*
Day 7	6.3 ± 0.6	3.6 ± 0.2*	6.8 ± 0.6	3.5 ± 0.3

(*n* = 7/group, except at day 7: *n* = 5 euglycemic and 2 hyperglycemic animals). ^∗^ *P* < 0.05 versus sham surgery.

**Table 2:** Number of foot necrosis after resection of the main limb arteries in euglycemic compared to hyperglycemic animals.

	Euglycemic animals		Hyperglycemic animals	
	Sham surgery	Resection	Sham surgery	Resection
*n*° of necrotic limbs (% of total *n*° of limbs)	0	2 (29)	0	5 (71)*

(*n* = 7/group). ^∗^ *P* < 0.05 versus euglycemic animals.
